# Association between phthalate exposure and accelerated bone maturation in Chinese girls with early puberty onset: a propensity score-matched case–control analysis

**DOI:** 10.1038/s41598-022-19470-4

**Published:** 2022-09-07

**Authors:** Shurong Huang, Zhe Su, Huiping Su, Yanhua Jiao, Qiru Su, Yao Yao, Li Zhou, Xiuxin Zheng, Xingliang Zhang

**Affiliations:** 1 School of Medicine, Southern University of Science and Technology, Shenzhen, 518055 Guangdong People’s Republic of China; 2 Department of Endocrinology, Shenzhen Children’s Hospital, Shenzhen, 518038 Guangdong People’s Republic of China; 3 Pediatrics Research Institute, Shenzhen Children’s Hospital, Shenzhen, 518038 Guangdong Province People’s Republic of China; 4 Central Laboratory, Longgang District Maternal and Child Healthcare Hospital, Shenzhen, Guangdong Province People’s Republic of China; 5 Shenzhen Center for Disease Control and Prevention, Shenzhen, 518055 Guangdong Province People’s Republic of China

**Keywords:** Medical research, Environmental chemistry

## Abstract

Estrogen can promote the acceleration of bone maturation and phthalate esters (PAEs) have estrogen-mimicking effects. We investigated whether PAEs are associated with the acceleration of bone age (BA) in girls with early onset of puberty (EOP). This case–control study enrolled 254 girls with EOP from the Endocrinology Department at Shenzhen Children’s Hospital between December 2018 and August 2019. Ultra-performance liquid chromatography and tandem mass spectrometry were used to analyze the 10 metabolites of PAEs (mPAEs) in urine samples. BA was measured using an artificial intelligence system. BA exceeding the chronological age (CA) by > 2 years (BA-CA ≥ 2 years) was referred to as significant BA advancement. Participants were divided into groups A (BA-CA ≥ 2 years; case group) and B (BA-CA < 2 years; control group). Propensity score matching (PSM) was performed for both groups in a 1:2 ratio with a caliper of 0.25. To identify potential dose–response relationships between PAEs exposure and BA advancement, we grouped the participants after PSM according to the tertiles of the mPAE concentrations. After PSM, 31 and 62 girls in groups A and B were selected. The concentration of Mono-ethyl phthalate (MEP) in group A was significantly higher than in group B (11.83 μg/g vs. 7.11 μg/g,* P* < 0.05); there was no significant difference in the levels of other mPAEs between the groups. The degree of BA advancement and proportion of significantly advanced BA in the lowest, middle, and highest tertiles of the MEP sequentially increased, as well as in the lowest, middle, and highest tertiles of Mono-(2-ethyl-5-carboxypentyl) phthalate; however, these were only statistically different between the highest and lowest MEP tertiles (both *P* < 0.05). For the remaining mPAEs, differences in the degree of BA advancement among the lowest, middle, and highest tertiles, as well as differences in the proportion of significantly advanced BA among the lowest, middle, and highest tertiles, were not significant (all *P* > 0.05). Our findings suggested that MEP was positively associated with BA advancement in girls with EOP. Exposure to PAEs may promote accelerated bone maturation.

## Introduction

Bone maturation, evaluated clinically by bone age (BA), refers to the developmental process from the emergence of ossification centers to the adult form of bones^1^. Acceleration of bone maturation manifests as a BA that exceeds the chronological age (CA), known as BA advancement. Significant BA advancement suggests an attenuation of the height growth potential. In other words, although children with advanced BA are taller than their peers during childhood, their growth plates fuse earlier and they may be shorter than their peers in adulthood. Shorter height may influence their physical and mental health from childhood to adulthood^2^; thus, significantly accelerated bone maturation, which could lead to impaired final adult height, is a social health issue that deserves attention. However, children with considerably advanced BA (BA-CA ≥ 2 years) have been observed in different populations: approximately 10% of healthy prepubescent Chinese children (unpublished observations by our research team); up to 21% of healthy children in the United States^3^; and 48.7% of girls with isolated premature thelarche, who should not have significantly advanced BA^4,5^. Moreover, a long-term trend of overall accelerated bone maturation in children has been observed in many countries^3,6–10^. Unfortunately, existing research results cannot fully explain the acceleration of bone maturation in children. Estradiol (E_2_) is the major factor that promotes bone maturation and growth plate fusion^11–15^. The body mass index (BMI), dehydroepiandrosterone sulfate (DHEAS), serum insulin-like growth factor-1 (IGF-1), and E_2_ are the most recognized independent risk factors for advanced bone maturation, explaining only 19.3–24% of all cases^4,16^, which indicates that more than half of the factors accelerating bone maturation remain unknown.

Phthalate esters (PAEs) are environmental endocrine-disrupting chemicals that interfere with the functioning of an organism’s endocrine system. Human exposure to PAEs is widespread and is a serious potential problem for human health, especially for children during the immature and developmental stages^17^. PAEs have been shown to have estrogenic effects^17–20^. PAEs exposure reportedly increases the incidence of precocious puberty in girls^21^. However, there is a lack of clinical case studies on the association between PAEs exposure and accelerated bone maturation. The impact of PAEs on accelerated bone maturation must be identified as soon as possible because PAEs are ubiquitous in the various ecosystems of the Pearl River Delta region in China, where Shenzhen is located^22–24^, indicating that children are almost continuously exposed to them.

In China, central precocious puberty (CPP)^25^ is defined as puberty before the age of 8 years while early puberty (EP)^26^ is defined as entering puberty between ages 8 and 9.5 years. Both CPP and EP present early functional initiation of the hypothalamic-pituitary–gonadal axis and BA advancement; however, definitions of the puberty onset age overlap and vary by country^26^. Therefore, in this study, we referred to CPP and EP together as the early onset of puberty (EOP). In this study, we investigated whether PAEs could accelerate bone maturation in girls with EOP. For our research purposes, we examined 10 urinary PAE metabolites (mPAEs) in the urine samples of girls with EOP and evaluated BA using emerging artificial intelligence (AI) technology to reduce manual evaluation errors. Propensity score matching (PSM) was used to balance confounding factors. Monoethyl phthalate (MEP) was positively associated with accelerated bone maturation.

## Methods

### Participants

Parents or guardians of all study participants provided written informed consent. All procedures were conducted according to the relevant guidelines and regulations. This study was approved by the Ethics Committee of the Shenzhen Children’s Hospital (201905503). We performed follow-ups with most girls aged 6–10 years with an early occurrence of secondary sexual characteristics in the outpatient department of our hospital. They were advised temporary hospitalization to complete gonadotropin-releasing hormone (GnRH) stimulation tests if they met the indications for hospitalization as follows: breast development occurring before the age of 9.5 years and persisting for at least 3 months, with a combination of at least one of the following: (1) the breast Tanner stage progressed in no more than 6 months; (2) BA exceeded CA for more than 2 years, and (3) accelerated height growth. Girls suspected of CPP or EP were verified via the GnRH stimulation test, and pathological causes, such as central nervous system tumors, were ruled out in the Department of Endocrinology at Shenzhen Children’s Hospital between December 2018 and August 2019.

The inclusion criteria were as follows: (1) breast development before 9.5 years old; (2) GnRH stimulation test results were positive: the ratio of the peak luteinizing hormone (LH) to the peak follicle-stimulating hormone (FSH) was > 0.6 while the level of the peak LH was > 5.0 IU/L; (3) pelvic ultrasound: the length of the uterus was > 3.4 cm, the volume of the ovary was > 1 mL, and there were more than two follicles with a diameter of > 4 mm; and (4) without other causes, such as a central nervous system tumor, peripheral precocious puberty, or local mammary gland hyperplasia, among others. Our study included 302 girls initially diagnosed with CPP or EP.

**Figure 1:**
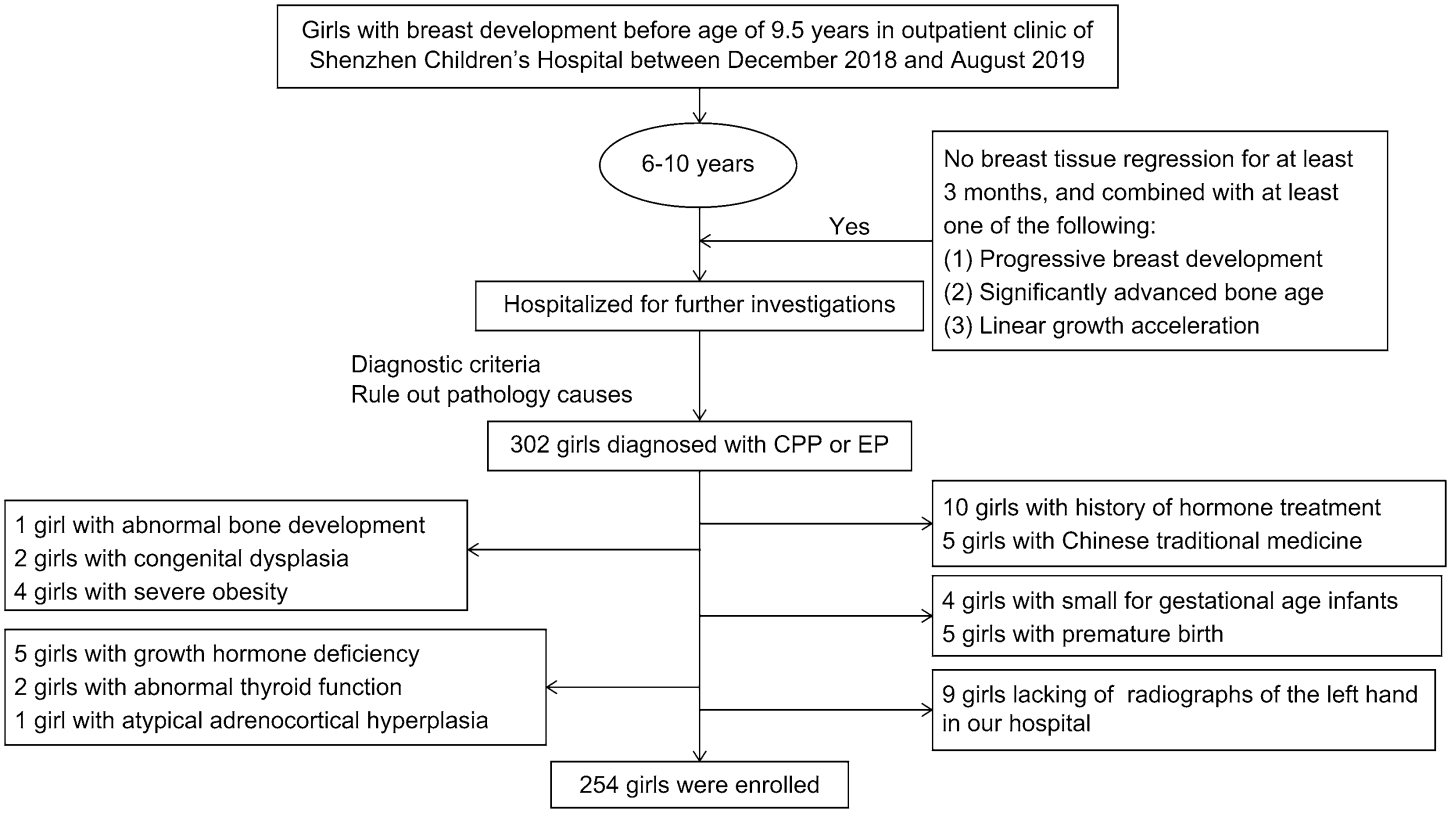
Flowchart of the population selected for the study.

Girls with a history of hormone treatment or Chinese traditional medicine, other endocrine diseases (including atypical adrenocortical hyperplasia, abnormal thyroid function, and growth hormone deficiency), abnormal bone development, congenital dysplasia, severe obesity, infants small for their gestational age, premature birth, and lack of left-hand radiographs in our hospital were excluded. Ultimately, 254 girls were enrolled in this study (Fig. 1).

This was a case–control study. The enrolled girls were divided into group A (BA-CA ≥ 2 years) as the case group and group B (BA-CA < 2 years) as the control group according to whether BA was significantly advanced. To identify potential dose–response relationships between BA advancement and mPAE concentrations, we additionally categorized the girls into groups according to the tertiles of the urinary mPAE concentration: lowest (< Q1), middle (Q1–Q2), and highest (> Q2) tertiles.

### Physical and laboratory examination

Standing height (cm) and body weight (kg) were measured using the same apparatus and following the same standard procedure. The BMI was calculated as the body weight (kg)/height (m)^2^. Breast development was staged by well-trained pediatricians from the endocrinology department according to the Tanner criteria.

GnRH stimulation tests and venous blood samples were collected to determine serum hormone levels between 8:00 and 10:00 am in a fasting state. The gonadotropin injection volume was 2.5–3.0 µg/kg; each injection volume did not exceed 100 µg in the GnRH stimulation test. The E_2,_ LH, and FSH levels were measured using immunochemiluminometric assays.

The BMI, DHEAS, and IGF-1 levels were standardized and expressed as standard deviation scores (SDS) according to reference data for different sexes and ages of Chinese children^27–29^, calculated as follows: (measured value − mean value for the same age and sex)/standard deviation.

### Urinary phthalate metabolite analysis

Polypropylene tubes were used to collect spot urine samples (50 mL). Urine samples were collected during the hospital stay and transported to the laboratory at the Shenzhen Center of Disease Control and Prevention on ice within 4 h, stored at − 40 °C, and tested within 1 year.

A total of 10 urinary mPAEs were detected in this study: monoethyl phthalate (MEP), mono-methyl phthalate (MMP), monobutyl phthalate (MBP), mono-isobutyl phthalate (MiBP), mono-benzyl phthalate (MBzP), mono (2-ethyl-5-hydroxyhexyl) phthalate (MEHHP), mono (2-ethylhexyl) phthalate (MEHP), mono (2-ethyl-5-carboxypentyl) phthalate (MEOHP), mono -(2-ethyl-5-carboxypentyl) phthalate (MECPP), and mono -[(2-carboxymethyl)hexyl] phthalate (MCMHP). We selected the 10 mPAEs because their parent compounds have been detected at relatively high levels in the Pearl River Delta region, where Shenzhen is located^23^; their detection rates are high in the urine of primary school students in Shenzhen^24^. All urinary mPAEs were analyzed via ultra-performance liquid chromatography and tandem mass spectrometry (UHPLC-MS/MS). Details of the method, including information on the relatively isotopically labeled internal standards and reagents, have been reported previously^24^. Urinary creatinine concentrations were determined using UHPLC-MS/MS to correct urine dilution^30^.

### BA assessment

BA was evaluated using an automated AI system (Yitu Healthcare, Hangzhou, China), known as the intelligent diagnosis system for child growth and development, which was developed for Chinese children using the TW3-Chinese RUS method^31^. The TW3-Chinese RUS method is a standard method for determining bone maturity in Chinese children based on the TW3 RUS method^32^.

### Statistical analysis

The detection threshold of E_2_ at our hospital was 20 pg/mL. The concentrations of E_2_ and mPAEs below the limit of detection (LOD) were replaced with 1/2 LOD. PAEs are often classified as low-molecular-weight (LMW) and high-molecular-weight (HMW) phthalates during exposure analyses^33,34^. ΣDi(2-ethylhexyl) phthalate (DEHP) is the sum of the creatinine-corrected concentrations of the five metabolites of DEHP (MCMHP, MEHP, MEOHP, MEHHP, and MECPP). ΣLMW is the sum of creatinine-corrected concentrations of the four LMW phthalate metabolites (MBP, MMP, MiBP, and MEP). ΣHMW is the sum of the creatinine-corrected concentrations of the six HMW phthalate metabolites (ΣDEHP and MBzP). ΣmPAE is the sum of the creatinine-corrected concentrations of all mPAEs (ΣLMW and ΣHMW)^35^.

Differences between the two groups of continuous variable data were analyzed using the Student’s t-test or Mann–Whitney U test. The differences between multiple groups of continuous variable data were tested using the Kruskal–Wallis one-way ANOVA. Rates between multiple groups were compared using the multiple-group chi-square test with Bonferroni correction. Statistical significance was set at *P* < 0.05. SPSS (version 23, IBM Corporation, Armonk, NY, USA) was used for data analysis.

PSM was performed on R software (version 3.3.3; R Foundation for Statistical Computing) using the MatchIt package (version 3.0.2). Based on previous research results and clinical experience, the study included the age of onset, disease duration, BMI SDS, DHEAS SDS, and IGF-1 SDS as covariates for the PSM^4,16^. The matching ratio of groups A and B was 1:2 because the number of patients in group B was 3.2-fold higher than in group A. Logistic regression was used to calculate the propensity scores. The nearest neighbor matching method was used for matching in PSM. To ensure a suitable sample size and better matching balance, after setting different caliper values several times for matching, the final caliper value was set to 0.25. The baseline variables of the two matched groups were compared; standardized mean differences were calculated to assess whether the included confounding factors were balanced.

### Ethics approval and consent to participate

All study participants provided written informed consent from their parents or guardians. This study was approved by the Ethics Committee of the Shenzhen Children’s Hospital (201905503).

## Results

### Baseline characteristics before and after PSM

After PSM, 93 girls (31 in group A and 62 in group B) were selected. Table 1 presents the baseline characteristics before and after PSM. There were no statistical differences in the baseline variables between groups A and B after PSM (*P* > 0.05). The standardized mean difference of the baseline variables after matching was < 0.1, indicating that the PSM balance was optimal. The median and quantile of E_2_ levels were 24.0 (10.0, 39.0) pg/mL in group A and 10.0 (10.0, 33.3) pg/mL in group B following PSM analysis, and no statistical differences between the two groups were found (*P* = 0.463).

**Table 1:** Baseline characteristics of girls before and after PSM analysis.

	Group A (After PSM)	Group B (After PSM)	*P* (After PSM)
Age at onset (years)	8.17 ± 0.90 (7.86 ± 0.94)	7.54 ± 0.84 (7.72 ± 0.8)	< 0.001 (> 0.05)
Course (month)	9.16 ± 7.40 (10.99 ± 9.57)	11.30 ± 8.78 (11.16 ± 8.80)	> 0.05 (> 0.05)
BMI SDS	0.50 ± 1.02 (0.85 ± 0.91)	1.01 ± 0.83 (0.83 ± 0.89)	< 0.001 (> 0.05)
IGF-1 SDS	0.34 ± 0.95 (0.60 ± 0.80)	1.10 ± 1.02 (0.56 ± 0.73)	< 0.001 (> 0.05)
DHEAS SDS	2.30 ± 2.98 (2.22 ± 3.52)	2.63 ± 2.73 (2.31 ± 2.60)	> 0.05 (> 0.05)

*PSM* propensity score matching, *BMI SDS* standard deviation score of the body mass index, *IGF-1 SDS* standard deviation score of the insulin-like growth factor-1, *DHEAS SDS* standard deviation score of dehydroepiandrosterone. Parentheses indicate after PSM.

### Urinary phthalate metabolites levels

The detection rates of mPAEs in the urine of the 93 girls were as follows: MBP (100%), MMP (98.9%), MECPP (98.9%), MiBP (96.7%), MEHHP (95.7%), MEP (92.5%), MEOHP (92.5%), MEHP (89.2%), MCMHP (86.0%), and MBzP (11.8%). The highest concentration of creatinine-corrected urinary mPAE was MBP (geometric mean: 203.77 μg/g), followed by MiBP (45.07 μg/g), MEP (8.55 μg/g), MMP (7.80 μg/g), MECPP (7.17 μg/g), MEHHP (5.99 μg/g), MEHP (5.10 μg/g), MEOHP (4.32 μg/g), MCMHP (2.58 μg/g), and MBzP (0.12 μg/g). The concentration of ΣLMW (308.41 μg/g) was higher than that of ΣHMW (28.05 μg/g). The concentration of MEP in group A was significantly higher than that in group B (11.83 μg/g vs. 7.11 μg/g, *P* < 0.05). There were no significant differences in the levels of the other urinary mPAEs, ΣDEHP, ΣLMW, ΣHMW, and ΣmPAEs between groups A and B (all *P* > 0.05) (Table 2).

**Table 2:** Urinary mPAE concentrations in girls from groups A and B post-PSM.

mPAEs (μg/g)	Group A (n = 31)	Group B (n = 62)
MMP	9.1 (4.00, 15.30)	7.84 (4.72, 12.00)
MEP	11.83 (6.96, 29.07)*	7.11 (3.32, 15.06)
MiBP	37.46 (24.63, 70.97)	33.95 (22.84, 74.49)
MBP	255 (142.29, 393.22)	225.4 (131.71, 377.83)
MECPP	7.56 (5.09, 14.3)	7.00 (4.37, 10.59)
MEHHP	5.71 (3.58, 13.71)	6.18 (3.54, 9.94)
MEOHP	4.44 (2.53, 9.96)	4.31 (2.41, 6.77)
MCMHP	3.13 (2.14, 4.82)	2.93 (1.72, 3.93)
MBzP	0.10 (0.05, 0.15)	0.12 (0.06, 0.18)
MEHP	3.74 (2.68, 9.46)	4.36 (2.82, 10.10)
ΣDEHP	26.58 (18.56, 58.96)	28.85 (16.41, 46.20)
ΣLMW	387.67 (198.06, 609.63)	287.71 (179.2, 601.43)
ΣHMW	26.71 (18.61, 59.06)	29.66 (16.46, 46.58)
ΣmPAEs	406.70 (210.00, 687.69)	314.63 (198.47, 659.97)

mPAE concentrations are described as a median (quartile). ΣDEHP: sum of creatinine-corrected concentrations of MCMHP, MEHP, MEOHP, MEHHP, and MECPP. ΣLMW: sum of creatinine-corrected concentrations of MBP, MMP, MiBP, and MEP. ΣHMW: sum of creatinine-corrected concentrations of ΣDEHP and MBzP. ΣmPAEs: sum of creatinine-corrected concentrations of all mPAEs.

*The concentration of urinary MEP in group A was significantly higher than that in group B (*P* < 0.05).

### Potential dose–response relationship between urinary phthalate metabolites concentrations and BA advancement

**Figure 2:**
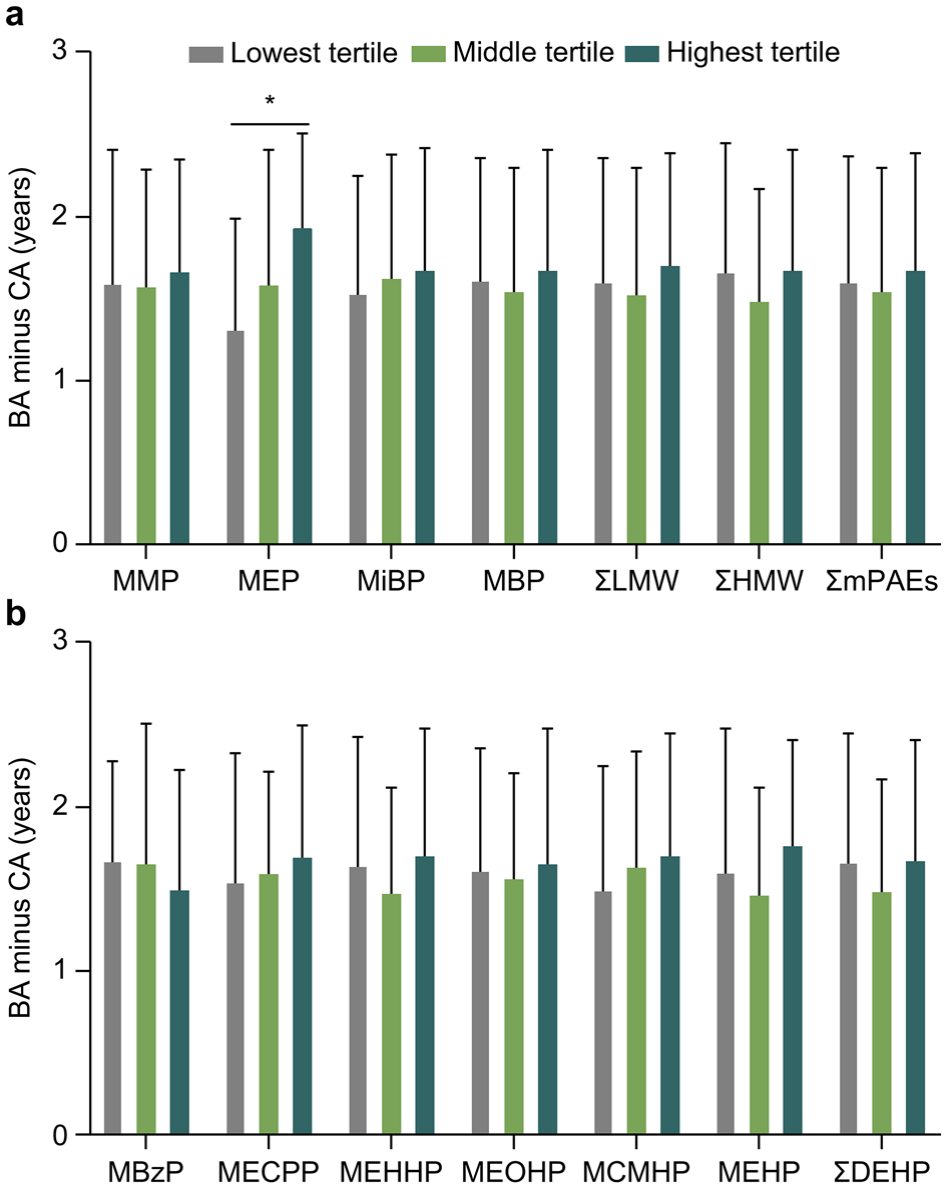
Bone age (BA) advancement in tertiles of the (**a**) low molecular weight mPAEs, ΣLMW, ΣHMW, and ΣmPAEs; and (**b**) high molecular weight mPAEs and ΣDEHP. The degree of BA advancement in the highest MEP tertile was statistically higher than that in the lowest MEP tertile (*P* < 0.05), as indicated by the asterisk (*). ΣDEHP: sum of the creatinine-corrected concentrations of MCMHP, MEHP, MEOHP, MEHHP, and MECPP. ΣLMW: sum of the creatinine-corrected concentrations of MBP, MMP, MiBP, and MEP. ΣHMW: sum of the creatinine-corrected concentrations of ΣDEHP and MBzP. ΣmPAEs: sum of the creatinine-corrected concentrations of all of the mPAEs.

**Figure 3:**
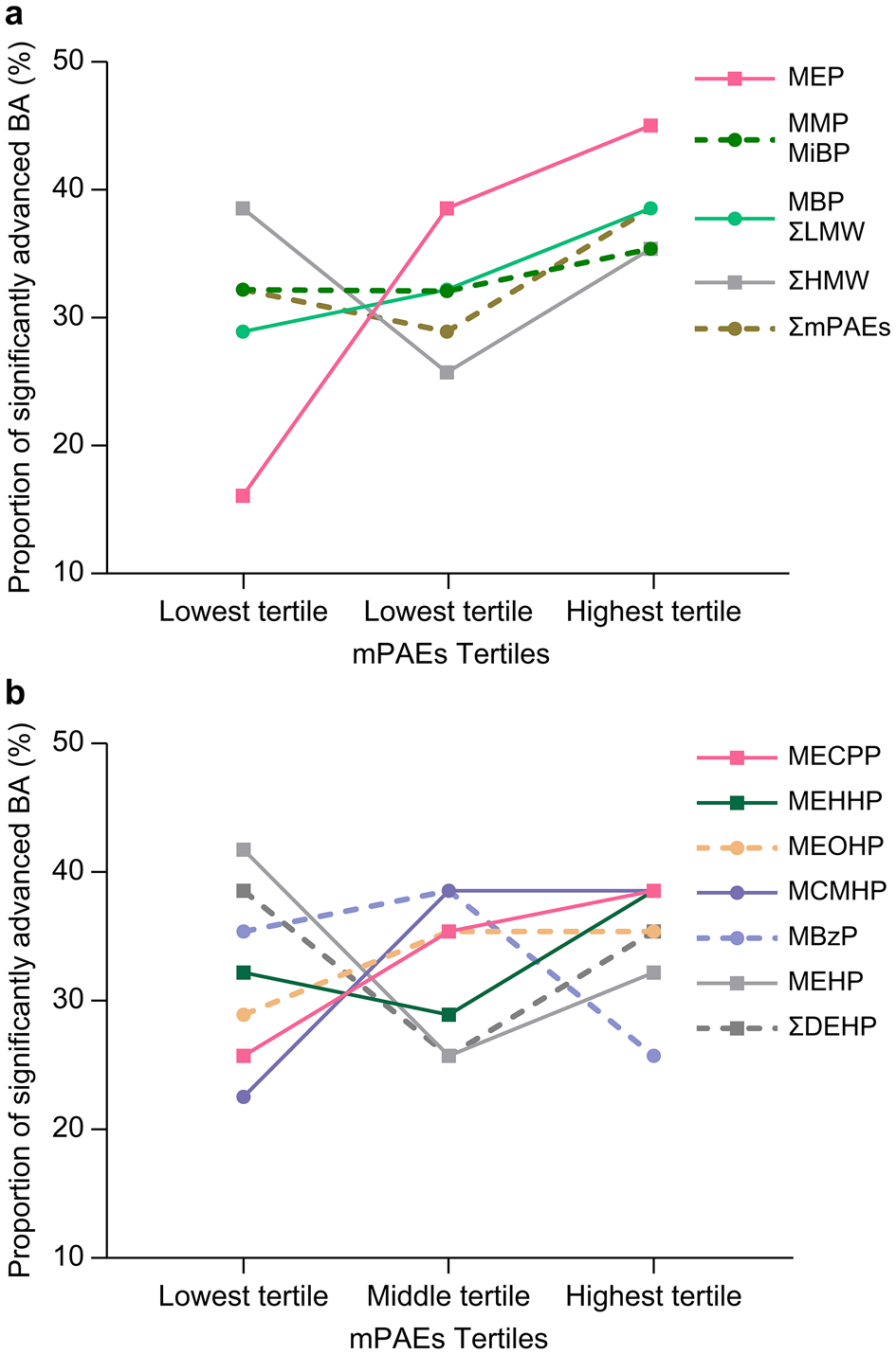
The proportion of significantly advanced bone age (BA) between the tertiles of the (**a**) low molecular weight mPAEs, ΣLMW, ΣHMW, and ΣmPAEs; and (**b**) high molecular weight mPAEs and ΣDEHP groups. The proportion of significantly advanced BA in the highest MEP tertile was statistically higher than that in the lowest MEP tertile (*P* < 0.05). ΣDEHP: sum of the creatinine-corrected concentrations of MCMHP, MEHP, MEOHP, MEHHP, and MECPP. ΣLMW: sum of the creatinine-corrected concentrations of MBP, MMP, MiBP, and MEP. ΣHMW: sum of the creatinine-corrected concentrations of ΣDEHP and MBzP. ΣmPAEs: sum of the creatinine-corrected concentrations of all of the mPAEs.

The differences in the degree of BA advancement (BA-CA) among the lowest, middle, and highest tertiles of each mPAE were analyzed and compared (Fig. 2), as were the differences in the proportion of significantly advanced BA among the lowest, middle, and highest tertiles of the mPAEs (Fig. 3). The degree of BA advancement and proportion of significantly advanced BA in the lowest, middle, and highest tertiles for MEP sequentially increased, as well as in the lowest, middle, and highest tertiles for MECPP (Figs. 2 and 3). However, the degree of BA advancement and proportion of significantly advanced BA were statistically different between the highest and lowest MEP tertiles (both *P* < 0.05) (Figs. 2 and 3). For the remaining urinary mPAEs, differences in the degree of BA advancement among the lowest, middle, and highest tertiles, as well as the differences in the proportion of significantly advanced BA among the lowest, middle, and highest tertiles, were not significant (all *P* > 0.05) (Figs. 2 and 3).

## Discussion

Bone maturation is affected by both internal and external factors^1^. However, the influence of environmental factors on bone maturation remains unclear. To our knowledge, the relationship between PAEs exposure and accelerated bone maturation has not yet been examined in girls with EOP. In this study, after using PSM to balance the confounding factors (i.e., disease duration, age of onset, BMI SDS, IGF-1 SDS, and DHEAS SDS) that may affect bone maturation^4,16^, we found that the urinary MEP concentration of girls with significantly advanced BA was significantly higher than that in the control group. The degree of BA advancement and proportion of significantly advanced BA in the highest MEP tertile were significantly higher than those in the lowest tertile group. Urinary metabolite concentrations can serve as a valuable method for assessing phthalate exposure in humans^30^. Therefore, our findings indicate that MEP, a monoester metabolite of diethyl phthalate (DEP), may be positively associated with accelerated bone maturation in girls with EOP. PAEs may be an important environmental factor affecting BA.

Previous case reports and studies have shown that estrogen and estrogen receptor α (ERα) is the main determinant of bone maturation and epiphyseal fusion^11,12,15^. Higher estrogen concentrations can accelerate bone maturation and stimulate fusion of the epiphyseal growth plate, leading to longitudinal growth termination^13–15^. However, children are not only influenced by their estrogen production, but also by substances in the environment that have estrogenic effects, such as PAEs.

Evidence from in vivo and in vitro studies suggests that DEP may exert estrogenic activity by directly acting on the ER and/or increasing estrogen synthesis by inducing aromatase gene expression^17–20^. DEP-treated female mice exhibited altered estrous cycle patterns, similar to animals with an increased endogenous estrogen load and corresponding uterine histological changes^18^. Furthermore, DEP promoted an increase in aromatase gene expression in ovarian cells via ERα both in vivo and in vitro^18^. Other PAEs also had estrogenic effects; however, DEP and DEHP showed better docking efficiency with ERα and estrogen receptor β (ER_β_) than other PAEs in molecular docking studies^36^. These findings provide a theoretical basis for our findings and may partially explain the fact that only MEP was positively associated with accelerated bone maturation in this study, despite the lower concentrations of MEP compared to MBP. In this study, we also found that MECPP, a metabolite of DEHP, showed a similar pattern as MEP, but the difference was not statistically significant. Nonetheless, we cannot categorically deny the possibility that MECPP may be associated with accelerated bone maturation as PAEs exposure may have synergistic effects^36^.

The effects of estrogen on longitudinal bone growth are biphasic. Low doses of estrogen promote bone elongation, while high doses induce growth plate senescence and epiphyseal fusion, resulting in the cessation of longitudinal bone growth^11^. The girls in this study had attained puberty and showed higher estrogen levels relative to prepubertal children. Whether DEP plays a role in accelerating bone maturation on this basis is unclear as we are currently unable to assess urinary DEP alone or all PAE levels corresponding to the amount of E_2_. This needs further evaluation through clinical and basic research.

DEP is as widely sourced as other PAEs; therefore, we are exposed to it almost constantly. Moreover, modern lifestyles may increase the risk of exposure to high doses of PAEs^37^. DEP is a colorless and odorless oily liquid that is soluble or partially miscible with organic molecules with aromatic properties^38^. Therefore, it is often used as a solvent, carrier, and cosmetic ingredient for fragrances and is widely used in personal care products, especially perfumes, skin care lotions, hair care products, cosmetics, and nail polishes, among others^37–39^. DEP is not chemically bound to the product; therefore, it can be released into the environment in a gas or particulate phase^37^. The use of perfumes is significantly associated with the concentration of DEP in indoor dust^37^. Skin exposure to DEP is the main route of exposure for the population, especially women who frequently use personal care products, whereas the inhalation of indoor air is the main route of exposure for children to DEP^39^. Additionally, DEP in the environment can enter the human body through ingestion via the digestive tract and skin directly from the air^30,40^.

This study found weak evidence that MEP exposure may be positively associated with accelerated bone maturation in girls with EOP. Future studies must therefore investigate the impact that PAEs exposure has on accelerated bone maturation and its underlying mechanism. At the same time, we should raise awareness about reducing exposure to PAEs, especially for children.

### Study limitations

One of the major limitations of this study was that a single spot urine test for mPAEs reflects only the short-term exposure level of PAEs. Changes in lifestyle, such as dietary habits and the use of nursing products, could also affect the results. Another major limitation was the small sample size (due to limited EOP cases), which reduces the statistical power and strength of the evidence.

## Conclusions

Our findings indicate that MEP could be positively associated with accelerated bone maturation in girls with EOP by detecting MEP concentrations in urine samples. We should raise awareness of the need to reduce exposure to PAEs. Future studies must examine the relationship between PAEs exposure and the acceleration of bone maturation.

## Data Availability

The datasets used and/or analyzed during the current study are available from the corresponding author on reasonable request.

## Abbreviations

AI: Artificial intelligence
BA: Bone age
BMI: Body mass index
CA: Chronological age
CPP: Central precocious puberty
DEHP: Di(2-ethylhexyl) phthalate
DEP: Diethyl phthalate
DHEAS: Dehydroepiandrosterone sulfate
E_2_: Estradiol
EOP: Early onset of puberty
ERα: Estrogen receptor α
ERβ: Estrogen receptor β
FSH: Follicle-stimulating hormone
GnRH: Gonadotropin-releasing hormone
HMW: High-molecular-weight
LH: Luteinizing hormone
LMW: Low-molecular-weight
LOD: Limit of detection
MBP: Monobutyl phthalate
MBzP: Mono-benzyl phthalate
MCMHP: Mono-[(2-carboxymethyl)hexyl] phthalate
MECPP: Mono-(2-ethyl-5-carboxypentyl) phthalate
MEHHP: Mono (2-ethyl-5-hydroxyhexyl) phthalate
MEHP: Mono (2-ethylhexyl) phthalate
MEOHP: Mono (2-ethyl-5-carboxypentyl) phthalate
MEP: Mono-ethyl phthalate
MiBP: Mono-isobutyl phthalate
MMP: Mono-methyl phthalate
mPAEs: Metabolites of PAEs
PAEs: Phthalate esters
PSM: Propensity score matching
SDS: Standard deviation scores
